# Massive Upper Gastrointestinal Bleeding

**DOI:** 10.21980/J8W93W

**Published:** 2022-01-15

**Authors:** Eytan Shtull-Leber, Amrita Vempati, Geoff Comp, Aneesh T Narang

**Affiliations:** * University of Arizona College of Medicine, Banner University Medical Center, Department of Emergency Medicine, Phoenix, AZ; ^ Creighton University School of Medicine Phoenix Program, Maricopa Medical Center, Department of Emergency Medicine, Phoenix, AZ

## Abstract

**Audience:**

This case is targeted to emergency medicine residents of all levels.

**Introduction:**

Upper gastrointestinal bleeding (UGIB) is a common chief complaint encountered in the emergency department, resulting in over 500,000 hospitalizations and 20,000 deaths annually in the United States.^1^ The diagnosis and management of UGIB in stable patients is typically fairly straightforward. However, there are a number of circumstances where the treatment of UGIB is much more challenging, and emergency medicine (EM) physicians should be familiar with, and have experience managing, these difficult presentations. Massive UGIB can necessitate the need for management of a difficult airway in the setting of airway contamination, as well as placement of a gastroesophageal balloon tamponade device. The appropriate use and indications for performing this high-risk/low-frequency procedure requires dedicated practice. Furthermore, the management of gastrointestinal hemorrhage in a patient with a religious objection to the administration of blood products, including Jehovah’s Witnesses, can be especially challenging and requires knowledge of alternative therapies to support blood pressure, oxygen carrying capacity, and decrease coagulopathy.^2^,^3^

**Educational Objectives:**

By the end of this simulation, learners will be able to: 1) manage a hypotensive patient with syncope and hematemesis, 2) pharmacologically manage an acute UGIB addressing the various causes, 3) recognize worsening clinical status and intervene by performing difficult airway management, 4) place a gastroesophageal balloon tamponade device.

**Educational Methods:**

This simulation was conducted with a high-fidelity mannequin with a separate medium-fidelity intubating mannequin that was modified to allow rapid filling of the oropharynx with simulated blood. Due to the COVID-19 pandemic, a total of six EM residents in various levels of training participated in the simulated patient encounter while the rest of the learners watched the simulation and participated in the debrief via video conference.

**Research Methods:**

Following the simulation and debrief session, all the residents, including those who participated in-person and via video conference, were sent a survey via surveymonkey.com to assess the educational quality of the simulation.

**Results:**

Overall residents expressed positive feedback on the scenario, noting that the case was realistic, appropriately complex, and improved their medical knowledge and procedural skills.

**Discussion:**

This case has a mixture of high-fidelity and medium-fidelity components which can be easily reproduced. The case was extremely useful in teaching EM residents of all levels not only how to manage large volume UGIB in a patient who is also a Jehovah’s Witness, but also how to manage the airway and place a gastroesophageal balloon tamponade device. The case starts with a patient presenting with syncope and as the case unfolds, the patient’s clinical status deteriorates, requiring learners to resuscitate, intubate, and obtain a gastroesophageal balloon tamponade.

Residents commented that managing this case of an UGIB was extremely challenging because it exposed and filled important gaps in both their knowledge and procedural skills. Residents struggled most with identifying alternative therapies to blood products in patients with religious objections, and the step-by-step process of placing a Blakemore tube.

**Topics:**

Upper gastrointestinal bleed, hemorrhagic shock, Jehovah’s Witness, difficult airway.

[Fig f1-jetem-7-1-s21]

**Figure f1-jetem-7-1-s21:**
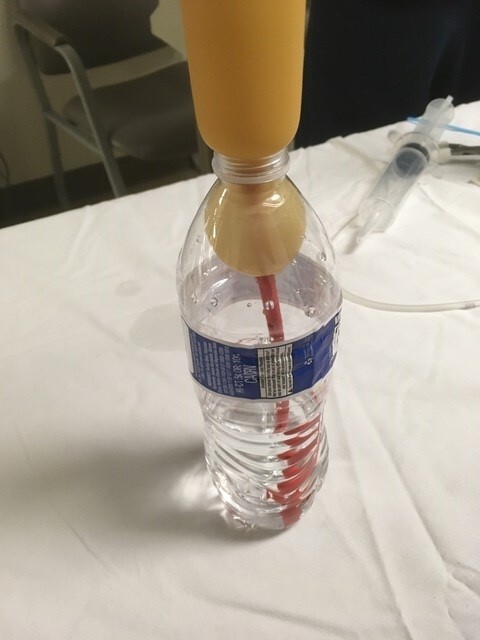


## USER GUIDE

**Table:** 

List of Resources:	
Abstract	21
User Guide	23
Instructor Materials	26
Operator Materials	36
Debriefing and Evaluation Pearls	41
Simulation Assessment	46

**Learner Audience:**

Interns, junior residents, senior residents

**Time Required for Implementation:**

Instructor Preparation: 30 minutes

Time for case: 20 minutes

Time for debriefing: 40 minutes

**Recommended Number of Learners per Instructor:**

4

**Topics:**

Upper gastrointestinal bleed, hemorrhagic shock, Jehovah’s Witness, difficult airway.

**Objectives:**

By the end of this simulation session, the learner will be able to:

1. Manage a hypotensive patient with syncope and hematemesis
2. Pharmacologically manage an acute UGIB addressing the various causes
3. Recognize worsening clinical status and intervene by performing difficult airway management
4. Place an esophageal balloon tamponade device

### Linked objectives and methods

The case begins with the patient presenting with syncope and a history of hematemesis and is found to be hypotensive. The learners will have to assess the patient and have a thoughtful approach to management (objective #1). They will have to get further history to assess for cirrhosis and arrive at esophageal varices as the likely etiology of the bleeding. Learners will need to give medications to address this cause (objective #2). The patient will continue to deteriorate due to variceal bleeding, pushing the learners to recognize the worsening clinical status. They will need to prepare for a difficult airway secondary to massive UGIB (objective #3). They will also need to place a gastroesophageal balloon tamponade device such as a Blakemore tube to further tamponade the bleeding (objective #4).

### Recommended pre-reading for instructor

We strongly recommend reviewing the following videos about the placement of the two most common gastro-esophageal balloon tamponade devices, in this order:

- Mason J. Overview of GI Tamponade Balloons. EM:RAP. https://www.emrap.org/hd/playlist/gastroPL/chapter/overviewofgi/overviewofgi. Published April 2016.^4^
- Mason J. Placement of a Minnesota Tube for Bleeding Varices. EM:RAP. https://www.emrap.org/hd/playlist/gastroPL/chapter/placementofa1/placementofa. Published April 2016.^5^
- Mason J. Placement of a Blakemore Tube for Bleeding Varices. EM:RAP. https://www.emrap.org/hd/playlist/gastroPL/chapter/placemenofa/placemenofa. Published April 2016.^6^

If the instructor is unfamiliar with the suction-assisted laryngoscopy and airway decontamination (SALAD) technique for intubation in the setting of massive hematemesis, we recommend reading this and watching the video:

- DuCanto J. SALAD. Open Airway. https://openairway.org/salad/. Published June 16, 2019.^7^

To review pharmacologic options for the management of bleeding in a patient who refuses blood transfusion due to religious objection, we recommend the following sources:

- Crookston K, Silvergleid A, Tirnauer J. The approach to the patient who declines blood transfusion. UpToDate. https://www.uptodate.com/contents/the-approach-to-the-patient-who-declines-blood-transfusion. Updated May 26, 2020.^2^
- Escobar, M. Hemostatic agents for Jehovah’s Witnesses. University of Texas Health Science Center at Houston. https://med.uth.edu/harrishealth/wp-content/uploads/sites/26/2015/01/Handout_of_hemostatic_agents_for_JW_3-8-14.pdf.^3^

### Results and tips for successful implementation

This simulation was designed for EM residents to improve their resuscitation skills by managing an extremely challenging case of massive UGIB that requires intubation and gastric balloon tamponade, in a patient who has a religious objection to blood product administration. The case was performed in a high-fidelity simulation setting using an additional medium-fidelity modified intubating mannequin to simulate airway decontamination and a low-fidelity model of a stomach and gastroesophageal junction to allow placement and visualization of a gastric balloon tamponade device.

To create realistic experience of an obscured airway and to practice the suction assisted laryngoscopy and airway decontamination (SALAD) technique,^7^ we created a medium-fidelity contaminated airway model (Video 1). The model consisted of a standard intubating mannequin with the lungs and stomach removed. Through the inferior gastroesophageal opening, a 7.5 endotracheal tube was inserted with the balloon inflated to create a seal, and the back end was connected via a christmas tree connector and pediatric ECMO tubing to a bag of watered-down washable red paint to simulate blood. Endotracheal tubes were also inserted with inflated balloons into the bilateral lung openings, also from an inferior approach, to prevent simulated blood from draining out of the oropharynx through the lungs. This allowed rapid filling of the oropharynx with contaminant that required aggressive suction to allow airway visualization.

In addition, we purchased an expired Blakemore tube from dotmed.com for $120. Either a Minnesota tube or a Blakemore tube can be used for tamponade. The difference between the two can be discussed during the debriefing session. We used a clear plastic water bottle as a low-fidelity model of a stomach, with the mouth of the water bottle representing the gastroesophageal junction, to demonstrate appropriate balloon placement (Image 1).

Some of the additional tips for successful implementation of this include:

- Create teams of 2–3 learners with mixed levels of training to balance experience levels with management of UGIBs and these complex procedures. Before the beginning of the case, allowing the team members to assign roles will help in running the case smoothly.
- The nursing cues can be given for more junior learners to help them through the case.
- The portion of the case with the Jehovah’s Witness component can be entirely removed for junior learners.

The case was designed and implemented during the 2020–2021 academic year, during the COVID-19 pandemic. It was piloted in a socially distanced manner in 3 separate sessions, each involving 2 residents participating live in the simulation center and 10 residents observing and participating in the debrief via video conference. All learners were from the same residency program and were partnered to balance their level of training.

Following the simulation and debrief session, participants, including those who participated in-person and via video conference (n=37), were provided a survey to assess the educational quality of the simulation and were also asked for open-ended feedback. There were 16 respondents (43% of participating residents), including 5/6 (83%) live participants, 8 (50%) interns, 6 (37.5%) PGY-2s, and 2 (12.5%) PGY-3s. Overall, residents expressed positive feedback on the scenario, noting that the case was realistic, appropriately complex, and improved their medical knowledge as well as their procedural skills with regard to difficult intubations and placement of a Blakemore tube (Table 1).

The most common suggestions for improvement surrounded hands-on experience with the Blakemore tube. One resident suggested having both a Minnesota and a Blakemore tube available in-person for comparison. While this would certainly improve understanding of the differences, these devices are expensive and difficult to obtain for teaching purposes. An alternative would be to purchase the same balloon type that is available clinically to your residents. Another suggestion was to save additional time for residents, to practice placing the Blakemore tube after the debrief so they could practice the techniques that were just taught. Given the complexity of the case and of Blakemore tube placement, it may be beneficial to use this opportunity to use spaced repetition and set up a Blakemore tube placement procedure lab the following week.

### Associated Materials

- https://youtu.be/wJK4idQlawM

### Disclosures

The authors have no financial disclosures. Specifically, none of the authors receive any financial benefit from the sale or use of DuCanto catheters, or from the educational materials that are produced by Dr. DuCanto, including the SALAD technique. Any large-bore suction catheter on the market could likely be used in place of the DuCanto branded catheter.

## Supplementary Information



## Figures and Tables

**Table t3-jetem-7-1-s21:** 

#1	Point of care glucose
#2	Complete blood count (CBC)
#3	Complete metabolic panel (CMP)
#4	Coagulation panel
#5	Beta human chorionic gonadotropin (Beta hcg)
#6	Chest radiograph (CXR)
#7	Computed tomography (CT) head without contrast

**Table t4-jetem-7-1-s21:** 

**Vitals:**	Heart Rate (HR) 105	Blood Pressure (BP) 105/65	Respiratory Rate (RR) 22
	Temperature (T) 37.0°C	Oxygen Saturation (O_2_Sat) 98%	

**Table t5-jetem-7-1-s21:** 

*Point of care glucose*	110mg/dL

**Table t6-jetem-7-1-s21:** 

*Complete blood count (CBC)*	
White blood count (WBC)	13.0 ×1000/mm^3^
Hemoglobin (Hgb)	8.0 g/dL
Hematocrit (HCT)	24.0%
Platelet (Plt)	67 ×1000/mm^3^

**Table t7-jetem-7-1-s21:** 

*Complete metabolic panel (CMP)*	
Sodium	124 mEq/L
Chloride	93 mEq/L
Potassium	4.2 mEq/L
Bicarbonate (HCO_3_)	17mEq/L
Blood Urea Nitrogen (BUN)	54 mg/dL
Creatine (Cr)	1.3 mg/dL
Glucose	110 mg/dL
Total bilirubin	3.4 mg/dL
Alkaline phosphatase	90 units/L
Aspartate aminotransferase (AST)	130 units/L
Alanine aminotransferase (ALT)	70 units/L
Albumin	2.5 g/dL
Total protein	4.0 g/dL

**Table t8-jetem-7-1-s21:** 

*Coagulation Panel*	
Prothrombin time (PT)	45 seconds
Partial thromboplastin time (PTT)	32 seconds
INR	2.4

**Table t9-jetem-7-1-s21:** 

*Beta human chorionic gonadotropin (Beta hcg)*	negative

**Figure f2-jetem-7-1-s21:**
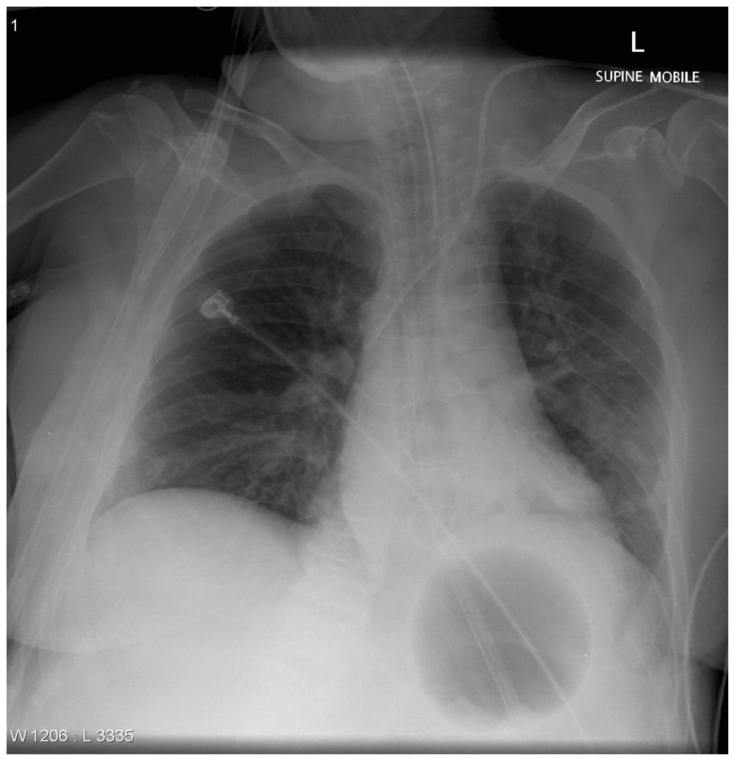


**Figure f3-jetem-7-1-s21:**
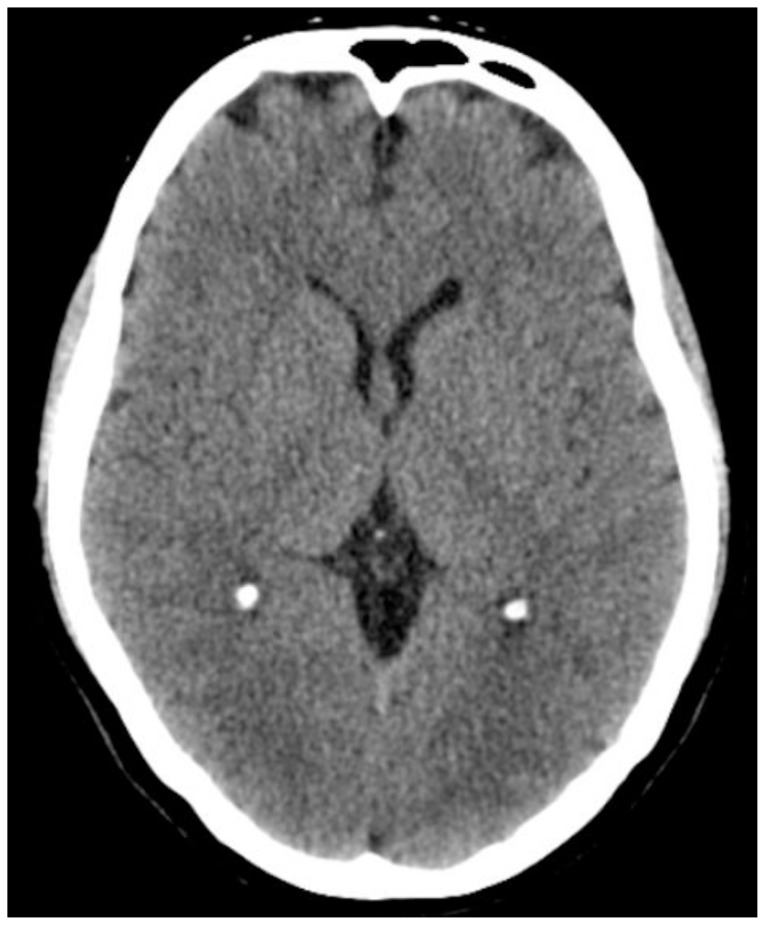


**Table t10-jetem-7-1-s21:** SIMULATION EVENTS TABLE:

Minute (state)	Participant action/trigger	Patient status (simulator response) & operator prompts	Monitor display (vital signs)
0:00 (Baseline)	Obtain brief history Obtain vital signs Attach patient to the cardiac monitor Obtain IV access Perform primary survey	Patient will have chief complaint of hematemesis and syncope Patient will be lying in bed, ill appearing and pale, but with normal primary survey	T 37.0 °C HR 105 BP 105/65 RR 22 O2 98%
1:00	Obtain a full history and physical exam	Patient moaning, holding her epigastric region She will have a history of “liver disease” and physical exam will reveal stigmata of cirrhosis	T 37.0 °C HR 105 BP 105/65 RR 22 O2 98%
3:00	Obtain large-bore IV access x2 (if not done) Order labs, CT head Start octreotide, pantoprazole, ceftriaxone	Prior to going to head CT, and regardless of medications ordered, patient will decompensate into hemorrhagic shock Nurse: “She is vomiting a lot of blood now,” [holds kidney basin with 500cc red bloody emesis] “and seems more altered.” Nurse: “Do you want me to send the patient to the CT scanner?”	T 37.0 °C HR 125 BP 85/40 RR 24 O2 94%
Call for uncrossmatched blood and airway equipment with difficult airway set-up	Nurse: “The patient’s husband is here.” Upon hearing the request for blood, patient’s husband: “My wife is a Jehovah’s Witness and cannot accept human-derived blood products.” Let the learners decide and specifically say what they want for airway (should plan for direct laryngoscopy with multiple large-bore suction catheters and “SALAD” technique If blood not called for, progress to 9:00 Status B	T 37.0 °C HR 125 BP 85/40 RR 24 O2 94%	Call for uncrossmatched blood and airway equipment with difficult airway set-up
7:00	Goals of care conversation regarding blood administration and intubation Initiate alternative treatments to blood products Consult GI	Patient’s husband will reiterate their objection to blood. Nurse will indicate that patient nodded in agreement Nurse: “Husband really doesn’t want blood, anything else we can give?” If vasopressors are started, blood pressure will improve. Massive hematemesis and altered mental status will continue. No immediate change will be seen with desmopressin, tranexamic acid, iron, erythropoietin. Recombinant factor VII is not available GI can be helpful in offering alternatives to blood products including desmopressin, tranexamic acid, iron, or erythropoietin If intubation is attempted before vasopressors, resuscitation and preparation proceed to status B. If given vasopressors and resuscitated first, proceed to status A	*With pressors* T 37.0 °C HR 110 BP 100/50 RR 24 O2 94% *Without pressors* T 37.0 °C HR 135 BP 80/35 RR 24 O2 94%
9:00 (Status A)	Intubate the patient with difficult airway plan and multiple suction setup	If intubated without aggressive suction setup, proceed to status B If participants resuscitate before they intubate, and use adequate suction, intubation will be successful. There will be no change in vitals.	*Successful intubation* T 37.0 °C HR 110 BP 100/50 RR 16 O2 100 %
9:00 (Status B)	Follow ACLS Perform CPR Give epinephrine Perform intubation	PEA arrest. Let the learners follow ACLS and give orders for resuscitation After intubation and 2 rounds of CPR and 1 epinephrine, patient will get ROSC.	*Failure to resuscitate or adequately suction* T 37.0 °C HR 0 BP 0/0 RR 0 O2 0 – 50%
12:00	Ventilator management Recognize the low blood pressure and need for placement of balloon tamponade device Place the device safely	Nurse: “The blood pressure is still low; do you think she is still bleeding?” Let the learners say what they exactly need for device placement. Consultants can be helpful in telling what they need, if needed. Nurse: “We only have a Blakemore tube [or whichever tube you have purchased] and this equipment. What else do you need?” If learners do not request/suggest Blakemore tube (or whichever tube you have purchased or is available at your hospital), patient will code, proceed to 9:00 Status B	T 37.0 °C HR 130’s BP 92/45 On ventilator
14:00	Request gastric balloon tamponade device (Blakemore or Minnesota tube) Obtain CXR after initial inflation of the gastric balloon	Appropriate placement, inflation, and tension of the gastric balloon will result in reduced hemorrhage via gastric port (and improved vitals), but there will be continued bleeding from the esophageal port (or OG tube, if Blakemore tube is used), necessitating pressure-monitored inflation of the esophageal balloon Facilitator prompt: how exactly do you determine if bleeding has decreased after inflation of the balloon? (Need to suction both stomach and esophagus) If learners do not place Blakemore tube (or whichever tube you have purchased or is available at your hospital) correctly, patient will code, proceed to 9:00 Status B	T 37.0 °C HR 100 BP 110/60 RR 16 O2 100 %
16:00	Obtain CT head Interpret the results Discuss with the husband Call consultants	Laboratory results will be given at this stage Patient will go to the CT scanner	
18:00 (Case completion)	Review CT head Discuss with consultants	CT head results Patient will be taken to endoscopy and admitted to the ICU afterwards. Interventional radiology can also be consulted	T 37.0 °C HR 100 BP 110/60 RR 16 O2 100 %

**Table t11-jetem-7-1-s21:** 

**Critical Actions:** Assess airway, breathing and circulation Connect the patient to the cardiopulmonary monitor and obtain large bore IV access Obtain a thorough history and perform a complete physical exam Assess for causes of UGIB Initiate pharmacologic therapy for UGIB from suspected varices including octreotide, ceftriaxone, proton pump inhibitor, and blood Order a CT head due to head trauma in a patient with alcoholic cirrhosis Monitor closely for worsening bleeding by frequent clinical assessments or placing a nasogastric tube Assess for airway protection in patient with worsening clinical status and establish endotracheal intubation Consider at least three alternatives to blood product administration for UGIB in patient who is a Jehovah’s Witness Place a gastroesophageal balloon tamponade device for massive UGIB to control the worsening bleeding Consult gastroenterology and admit the patient to the intensive care unit (ICU) Discuss the case with patient’s husband	0:00

**Table t12-jetem-7-1-s21:** 

	Milestone	Did not achieve level 1	Level 1	Level 2	Level 3
1	**Emergency Stabilization (PC1)**	□ Did not achieve Level 1	□ Recognizes abnormal vital signs	□ Recognizes an unstable patient, requiring intervention Performs primary assessment Discerns data to formulate a diagnostic impression/plan	□ Manages and prioritizes critical actions in a critically ill patient Reassesses after implementing a stabilizing intervention
2	**Performance of focused history and physical (PC2)**	□ Did not achieve Level 1	□ Performs a reliable, comprehensive history and physical exam	□ Performs and communicates a focused history and physical exam based on chief complaint and urgent issues	□ Prioritizes essential components of history and physical exam given dynamic circumstances
3	**Diagnostic studies (PC3)**	□ Did not achieve Level 1	□ Determines the necessity of diagnostic studies	□ Orders appropriate diagnostic studies. Performs appropriate bedside diagnostic studies/procedures	□ Prioritizes essential testing Interprets results of diagnostic studies Reviews risks, benefits, contraindications, and alternatives to a diagnostic study or procedure
4	**Diagnosis (PC4)**	□ Did not achieve Level 1	□ Considers a list of potential diagnoses	□ Considers an appropriate list of potential diagnosis May or may not make correct diagnosis	□ Makes the appropriate diagnosis Considers other potential diagnoses, avoiding premature closure
5	**Pharmacotherapy (PC5)**	□ Did not achieve Level 1	□ Asks patient for drug allergies	□ Selects an medication for therapeutic intervention, consider potential adverse effects	□ Selects the most appropriate medication and understands mechanism of action, effect, and potential side effects Considers and recognizes drug-drug interactions
6	**Observation and reassessment (PC6)**	□ Did not achieve Level 1	□ Reevaluates patient at least one time during case	□ Reevaluates patient after most therapeutic interventions	□ Consistently evaluates the effectiveness of therapies at appropriate intervals
7	**Disposition (PC7)**	□ Did not achieve Level 1	□ Appropriately selects whether to admit or discharge the patient	□ Appropriately selects whether to admit or discharge Involves the expertise of some of the appropriate specialists	□ Educates the patient appropriately about their disposition Assigns patient to an appropriate level of care (ICU/Tele/Floor) Involves expertise of all appropriate specialists
9	**General Approach to Procedures (PC9)**	□ Did not achieve Level 1	□ Identifies pertinent anatomy and physiology for a procedure Uses appropriate Universal Precautions	□ Obtains informed consent Knows indications, contraindications, anatomic landmarks, equipment, anesthetic and procedural technique, and potential complications for common ED procedures	□ Determines a back-up strategy if initial attempts are unsuccessful Correctly interprets results of diagnostic procedure
20	**Professional Values (PROF1)**	□ Did not achieve Level 1	□ Demonstrates caring, honest behavior	□ Exhibits compassion, respect, sensitivity and responsiveness	□ Develops alternative care plans when patients’ personal beliefs and decisions preclude standard care
22	**Patient centered communication (ICS1)**	□ Did not achieve level 1	□ Establishes rapport and demonstrates empathy to patient (and family) Listens effectively	□ Elicits patient’s reason for seeking health care	□ Manages patient expectations in a manner that minimizes potential for stress, conflict, and misunderstanding. Effectively communicates with vulnerable populations, (at risk patients and families)
23	**Team management (ICS2)**	□ Did not achieve level 1	□ Recognizes other members of the patient care team during case (nurse, techs)	□ Communicates pertinent information to other healthcare colleagues	□ Communicates a clear, succinct, and appropriate handoff with specialists and other colleagues Communicates effectively with ancillary staff

**Table 1:** Representative Learner Feedback on UGIB Simulation session (PGY, Post-Graduate Year)

Feedback	Year
“Only thing I would add is let the residents try and set up and place the Blakemore tube after the debriefing session…Overall, one of my favorite sim cases.”	PGY-2
“I really appreciated the flow of the case. I thought the complexity was excellent and I felt challenged during the case.”	PGY-1
“Excellent case. Lots of things I didn’t know that I didn’t know.”	PGY-2
“The case very clearly exposed a gap in our knowledge on how to perform this high risk, low frequency procedure. I now have a much better understanding on how to [insert a Blakemore tube] thanks to the simulation.”	PGY-2
“About as hard as you can make a GIB case:”	PGY-2
“Being able to see the Minnesota tube at the same time as the Blakemore would be helpful so we could really compare and contrast.”	PGY-1

## INSTRUCTOR MATERIALS

**Case Title:** Massive Upper Gastrointestinal Bleeding

**Case Description & Diagnosis (short synopsis):** A 46-year-old female with alcoholic cirrhosis presents with UGIB. Her vitals are initially borderline hypotensive, but she is ill-appearing and had a syncopal event this morning. While in the emergency department, she has an episode of massive bright red hematemesis and develops hemorrhagic shock. As the case progresses, the patient’s husband informs the providers that she is a Jehovah’s Witness. The patient will become more altered and continue to bleed requiring intubation and placement of balloon tamponade. The patient will need to be admitted to the intensive care unit (ICU) with the diagnosis of upper gastrointestinal bleeding secondary to esophageal varices.

**Equipment or Props Needed:**

Moulage for bruise on patient’s forehead, spider angioma on abdomen, and scleral icterus

Basin with red “blood”

High-fidelity mannequin with monitor

Medium-fidelity contaminated airway model using a modified intubating mannequin

Low-fidelity model of gastroesophageal junction using a clear plastic water bottle

Multiple suction catheters, including at least one large-bore catheter (eg, DuCanto catheter)

Working suction cannisters

Airway supplies:

- Nasal cannula
- Non-rebreather mask
- Laryngoscope and blades
- Endotracheal tube (ET) and stylet
- Bag-valve mask

IV supplies:

- 2 18-gauge angiocatheters
- IV tubing

Medications

- Octreotide
- Pantoprazole
- Norepinephrine
- Desmopressin
- Tranexamic acid
- Etomidate
- Succinylcholine
- Rocuronium
- Packed red blood cells

Balloon tamponade equipment

- Blakemore or Minnesota tube
- Ice water bucket
- Lubricant
- Posey sphygmomanometer
- Kelly clamps x3
- Luer-locked 50–60cc syringe x1
- Toomey 50–60cc syringe x1
- 3-way stop cocks x2
- Suction tubing
- Christmas tree connector
- Roll of kerlix
- 1L normal saline
- IV pole
- Pediatric bougie
- (If using a Blakemore tube): OG tube, permanent marker

**Confederates needed:**

Primary nurse

Patient’s husband, who can be played by the simulation operator

**Stimulus Inventory:** [Table t3-jetem-7-1-s21]

**Background and brief information:** A 46-year-old female brought in by her husband to the ED at a tertiary care center for “passing out” and “vomiting blood.”

**Initial presentation:** The patient is brought from home by her husband via private vehicle for hematemesis and syncope. The patient is ill-appearing but is able to answer questions. She is borderline hypotensive, tachycardic and tachypneic.

**How the scene unfolds:** A 46-year-old female is brought in from home by her husband for hematemesis and syncope. She had 2 episodes of 2 tablespoons of bright red hematemesis this morning and then became lightheaded and had a syncopal event just prior to arrival, prompting her visit to the ED. Learners should recognize stigmata of possible liver disease (lower extremity edema, scleral icterus) and should be concerned about potential for rapid decompensation. She should immediately be placed on the monitor, given 2 large-bore IVs, and started on octreotide, ceftriaxone, and a proton pump inhibitor for suspected UGIB. The dosing of the medications can be given by the learners or they may defer to pharmacy. Additional history should reveal a history of alcohol abuse, liver disease, and no blood thinners. If collateral history is obtained from the husband, he should not inform them of their religious objection to blood products unless asked specifically. He will offer this information voluntarily as soon as blood products are ordered.

Laboratory studies (point of care glucose, CBC, CMP, PT/INR, PTT, Beta-hCG, type and screen) should be ordered. A CT head without contrast should also be ordered to evaluate for subdural hematoma in the setting of head trauma (forehead bruising) in a patient who is a chronic alcoholic, but the patient will not be stable enough to go to radiology until after intubation and gastroesophageal balloon tamponade.

Regardless of initial actions taken by learners, the patient will develop massive hematemesis in the ED and become more altered, suggesting decompensation into hemorrhagic shock. Learners should call for uncrossmatched (O negative) blood and prepare for intubation. Upon hearing the call for blood, the patient’s husband will inform the learners that she is a Jehovah’s Witness and has religious objections to all human-derived blood products. Learners should consider alternative therapy options including vasopressors, desmopressin, tranexamic acid, recombinant factor VII, iron, erythropoietin, and early placement of a gastroesophageal balloon tamponade device.

The simulation nurse will inform the learners of recurrent massive hematemesis and altered mental status, which should prompt the learners to prepare for intubation to protect the patient’s airway. Their plan should account for the patient’s mechanically difficult airway secondary to obstructed views from massive hematemesis, as well as her physiologically difficult airway secondary to hypotension. They should pre-oxygenate in the upright position, improve the blood pressure prior to intubation with vasopressors, utilize direct laryngoscopy, multiple large-bore suction catheters, and utilize the suction-assisted laryngoscopy and airway decontamination (SALAD) technique to visualize the vocal cords.

As a result of the patient’s recurrent massive hematemesis prior to intubation, as well as the limitations on blood product resuscitation, learners should recognize the need for rapid control of bleeding and should consider gastric balloon tamponade using a Blakemore or Minnesota tube (whichever is available in your hospital). Failure to confirm gastric balloon placement with chest radiography prior to full insufflation of the gastric balloon, or insufflation of the esophageal balloon without pressure monitoring, will result in rapid decompensation from esophageal rupture. A correctly placed balloon tamponade device will result in improvement of vital signs and bleeding. Gastroenterology, interventional radiology, and the intensive care unit (ICU) should all be consulted. A head CT should be obtained prior to transport to the ICU to evaluate for subdural hemorrhage now that the patient is stabilized.

**Critical actions:**

1. Assess airway, breathing and circulation
2. Connect the patient to the cardiopulmonary monitor and obtain large bore IV access
3. Obtain a thorough history and perform a complete physical exam
4. Assess for causes of UGIB
5. Initiate pharmacologic therapy for UGIB from suspected varices including octreotide, ceftriaxone, proton pump inhibitor, and blood
6. Order a CT head due to head trauma in a patient with alcoholic cirrhosis
7. Monitor closely for worsening bleeding by frequent clinical assessments or placing a nasogastric tube
8. Assess for airway protection in patient with worsening clinical status and establish endotracheal intubation
9. Consider at least three alternatives to blood product administration for UGIB in patient who is a Jehovah’s Witness
10. Place a gastroesophageal balloon tamponade device for massive UGIB to control the worsening bleeding
11. Consult gastroenterology and admit the patient to the intensive care unit (ICU)
12. Discuss the case with patient’s husband

**Case Title:** Massive Upper Gastrointestinal Bleeding

**Chief Complaint:** “vomiting blood” and “passed out”[Table t4-jetem-7-1-s21]

**General Appearance:** 46-year-old female, ill-appearing and pale, but awake and oriented times three.

**Primary Survey:**

- **Airway:** patent
- **Breathing:** clear to osculation bilaterally
- **Circulation:** 2+ radial pulses bilaterally

**History:**

- **History of present illness:** She had two episodes of two tablespoons of bright red hematemesis this morning and then became lightheaded and had a syncopal episode just prior to arrival, prompting her visit to the ED. She had one episode of melena yesterday. She denies chest pain, bleeding disorders, or being on blood thinners. If asked specifically, she drinks alcohol daily and has a history of “liver disease” but doesn’t know anything more about it.
- **Past medical history:** “Liver disease,” “heart disease,” and “kidney disease”
- **Past surgical history:** None
- **Patient’s medications:** “two pee pills” (furosemide and spironolactone), and “a poop pill” (lactulose)
- **Allergies:** No known drug allergies
- **Social history:** Daily alcohol consumption, Jehovah’s Witness (only if asked)
- **Family history:** no bleeding disorders

**Secondary Survey/Physical Examination:**

- **General appearance:** ill-appearing and pale, but awake and oriented
- **HEENT:**
  ○ **Head:** bruise to forehead
  ○ **Eyes:** scleral icterus
  ○ **Ears:** within normal limits
  ○ **Nose:** within normal limits
  ○ **Throat:** small amount of fresh blood in the oropharynx
- **Neck:** within normal limits
- **Heart:** regular rhythm with mild tachycardia, no murmurs
- **Lungs:** within normal limits
- **Abdominal/GI:** mildly distended, with spider angioma, soft, non-tender
- **Genitourinary:** within normal limits
- **Rectal:** gross melena
- **Extremities:** 1+ bilateral lower extremity edema
- **Back:** within normal limits
- **Neuro:** awake and oriented, though slightly slow to respond. Cranial nerves II-XII grossly intact. Strength 5/5 in all 4 extremities with equal sensation to light touch. No ataxia. No asterixis.
- **Skin:** pale, spider angioma on abdomen
- **Lymph:** within normal limits
- **Psych:** within normal limits

**Results:** [Table t5-jetem-7-1-s21] [Table t6-jetem-7-1-s21] [Table t7-jetem-7-1-s21] [Table t8-jetem-7-1-s21] [Table t9-jetem-7-1-s21]

*Chest radiograph (CXR)*

Gaillard F. Sengstaken-Blakemore tube. In: Radiopedia. https://radiopaedia.org/cases/sengstaken-blakemore-tube. CC BY-NC-SA 3.0.[Fig f2-jetem-7-1-s21]

*Computed tomography (CT) head without contrast*

Cuete D. Normal CT Brain. In: Radiopedia. https://radiopaedia.org/cases/normal-ct-brain. CC BY-NC-SA 3.0.[Fig f3-jetem-7-1-s21]

## OPERATOR MATERIALS

[Table t10-jetem-7-1-s21]

**Diagnosis:**

Massive upper gastrointestinal bleed secondary to ruptured esophageal varices

**Disposition:**

Transfer to endoscopy or Interventional Radiology (IR) suite followed by admission to the intensive care unit.

## DEBRIEFING AND EVALUATION PEARLS

### Massive Upper Gastrointestinal Bleeding in a Jehovah’s Witness

The diagnosis of UGIB is typically fairly straightforward, and is suggested by hematemesis and melena, but should also be suspected in unstable patients with massive hematochezia. A higher index of suspicion for occult UGIB needs to be maintained as an underlying cause of generalized weakness, dizziness, and syncope. Etiologies of UGIB are extensive and include esophageal varices, peptic ulcers, esophagitis, gastritis, and vascular malformations.

#### General treatment of Upper GI Bleeding

- Proton pump inhibitors are typically administered for all patients with UGIB. There is no mortality benefit, but they have been shown to reduce endoscopic stigmata of recent hemorrhage and need for endoscopic intervention.^8^ This is generally given as pantoprazole 80mg IV bolus. A subsequent drip has not been shown to be beneficial.
- Octreotide causes splanchnic vasoconstriction, which leads to a modest decrease in bleeding in the setting of variceal UGIB.^9^ There is no known mortality benefit. Octreotide is generally given to all patients with cirrhosis and an UGIB as a bolus of 50 mcg IV followed by a drip at 50 mcg/hr.
- Antibiotics are the only pharmacologic therapy that have been shown to have mortality benefit for UGIB in cirrhotic patients.^10^ This is felt to be due to decreased bacterial gut translocation and prevention of spontaneous bacterial peritonitis. Typically, a third-generation cephalosporin is administered.
- Blood transfusion is reserved for acute blood loss anemia and hemodynamically significant active bleeding, regardless of the hemoglobin level.
- Definitive treatment includes endoscopic banding, cautery, and injection, as well as endovascular embolization and the trans-jugular intrahepatic portosystemic shunt (TIPS) procedure.

#### Management of hemorrhage in patients who are Jehovah’s Witnesses

The religious objection to human-derived blood product administration amongst Jehovah’s Witnesses stems from a belief that it may affect their eternal salvation. As such, this objection is often fervent, and alternative therapies may be needed.^2^,^3^

- Recombinant factors VII, VIII, IX are available and are made by cloning the human factor gene and growing it in culture media without human protein or serum, and are therefore acceptable for use by Jehovah’s Witnesses. Evidence is scarce and these therapies are likely expensive and not always available.
- Desmopressin (DDAVP) can be used to release endogenous von Willebrand factor and factor VIII, and can improve platelet dysfunction, which may be especially useful if bleeding is felt to be, at least partially, uremic or related to NSAIDS or other platelet inhibitors.
- Vasopressors and IV fluids can be used to help support blood pressure, and may be necessary, but they will not increase oxygen carrying capacity, and IV fluids may actually be harmful due to dilution.
- Tranexamic acid (TXA) is useful in the treatment of traumatic and post-partum hemorrhage; however recent high-quality evidence (HALT-IT trial) suggests no mortality benefit in the setting of GIB, and there is an increased risk of venous thromboembolism and seizures.^11^ These complications were still quite rare (<1%), and shared decision making may be appropriate in this case, where other therapies are limited, but in general, TXA is not recommended for GIB treatment.
- Iron infusion and erythropoietin will help the patient regenerate additional red blood cells if they survive the initial UGIB. These medications will not help acutely and likely do not need to be administered in the ED.
- Early hemorrhage control is perhaps the most critical intervention in patients who object to blood transfusions. When both blood pressure and oxygen carrying capacity cannot be simultaneously supported with blood, early definitive therapy with endoscopic or endovascular interventions may be needed, even in a stable patient. In an unstable patient, early gastro-esophageal balloon tamponade may be life-saving.

#### Contaminated Airway Management

Intubating a contaminated airway with vomitus or blood can be extremely challenging. Pre-oxygenation can be nearly impossible, visualization of the vocal cords and other airway structures can be severely limited, and aspiration is extremely common. Video laryngoscopes tend to be obscured by airway contaminate, and even direct laryngoscopes can have their light source diminished by contact with opaque substances in the airway. Traditional suction devices, like the Yankhauer, are often inadequate due to their limited suction capacity and tendency to be easily clogged and rendered useless by chunky emesis or even blood clots. Techniques to decontaminate the airway, improve visualization, and increase the chances of first-pass intubation success include:

- Direct laryngoscopy is generally recommended over video laryngoscopy, which is commonly obscured by airway contaminate.
- The upright position should be used for pre-oxygenation and intubation where possible to decrease the risk of aspiration.
- Multiple suction catheters as well as large-bore suction devices, where available, should be used to improve the rate of airway decontamination. One of these can be placed and left in the esophagus for continuous suction.
- The suction-assisted laryngoscopy and airway decontamination (SALAD) technique is a method of laryngoscopy designed specifically for management of the vomit-filled airway.^7^ It involves leading with a large-bore suction device, followed by a direct laryngoscope. The suction device is then positioned in the esophagus for continuous suction, while it is shifted to the left of the patient’s mouth and held in place by the left border of the laryngoscope. This leaves the right side of the oropharynx open and cleared for tube delivery and additional suction as needed. A video demonstration of this technique is listed below.
- Airway adjuncts are also useful to have available. These include a bougie, intubating LMA (laryngeal mask airway), and a cricothyroidotomy kit, should airway contaminate be so severe that a “can’t intubate, can’t ventilate” situation arises.

#### Gastroesophageal Balloon Tamponade Devices

Gastroesophageal balloon tamponade devices, including the Blakemore and Minnesota tubes, are designed to temporize variceal upper GI bleeding by applying direct pressure to bleeding gastric and esophageal varices. The Blakemore and Minnesota tubes each have a gastric and esophageal balloon that can be inflated separately to tamponade adjacent bleeding varices, as well as a gastric section port at the distal tip of the tube. The Minnesota tube is a modified version of the Blakemore tube that has an additional suction port on top of the esophageal balloon, obviating the need for placement of an OG tube alongside the Blakemore tube to assess for persistent esophageal bleeding after inflation of the gastric balloon. Another important difference between the two devices is that the Minnesota tube’s gastric balloon accommodates significantly more air (500 cc versus 150 cc in the Blakemore tube).

The traditional indication for use of a gastroesophageal balloon tamponade device was to temporize a severe, life-threatening, suspected variceal bleed when definitive (endoscopic or endovascular) therapy is delayed. This is likely due to the high complication rate of balloon placement. However, when unable to stabilize a patient with blood transfusions (due to religious objection or resource availability), early control of bleeding is of critical importance and should prompt consideration of early balloon placement. Additionally, as has recently been pointed out in a March 2021 episode of EM:RAP, there are a number of steps that can be taken to significantly decrease the complication rate of balloon placement, making this procedure substantially safer than original reports.^12^ As such, placement of these devices can likely be considered much earlier, when the patient is beginning to become unstable, well before they are peri-arrest. These steps include intubating the patient prior to device placement to prevent asphyxiation from accidental oropharyngeal balloon inflation, obtaining an x-ray prior to fully inflating the gastric balloon to ensure it is not in the esophagus, and only inflating the esophageal balloon when necessary and when a manometer can be used to prevent over-inflation and esophageal rupture.

The insertion of a Blakemore or Minnesota tube is best explained in video format.^4^–^6^,^13^ Below are a number of videos that review how these devices should be used. However, the actual insertion of the device through the oropharynx and into the esophagus and stomach is much easier said than done. Some tricks to improve device placement include stiffening up the tube by soaking it in ice water (usually while performing endotracheal intubation) prior to use, or using the back end of a pediatric bougie placed into the most proximal of the gastric aspiration ports.^13^

#### Wrap Up

Here are some excellent videos summarizing the differences between the gastroesophageal balloon tamponade devices on the market and how to place the two devices you are most likely to have in your hospital. The last video demonstrates the bougie-assisted placement technique. We recommend watching the videos in this order:

- Mason J. Overview of GI Tamponade Balloons. EM:RAP. https://www.emrap.org/hd/playlist/gastroPL/chapter/overviewofgi/overviewofgi. Published April 2016.
- Mason J. Placement of a Minnesota Tube for Bleeding Varices. EM:RAP. https://www.emrap.org/hd/playlist/gastroPL/chapter/placementofa1/placementofa. Published April 2016.
- Mason J. Placement of a Blakemore Tube for Bleeding Varices. EM:RAP. https://www.emrap.org/hd/playlist/gastroPL/chapter/placemenofa/placemenofa. Published April 2016.
- Whitford B, Hinckley B. The Whitford Technique: Bougie-Aided Minnesota Tube Placement. YouTube. https://www.youtube.com/watch?v=BjyhGg1vBu0. Published May 2018.

To review the suction-assisted laryngoscopy and airway decontamination (SALAD) technique for intubation in the setting of massive hematemesis, we recommend reading this and watching the video:

- DuCanto J. SALAD. Open Airway. https://openairway.org/salad/. Published June 16, 2019.

To review pharmacologic options for the management of bleeding in a patient who refuses blood transfusion due to religious objection, we recommend the following sources:

- Crookston K, Silvergleid A, Tirnauer J. The approach to the patient who declines blood transfusion. UpToDate. Updated May 26, 2020. https://www.uptodate.com/contents/the-approach-to-the-patient-who-declines-blood-transfusion
- Escobar, M. Hemostatic agents for Jehovah’s Witnesses. University of Texas Health Science Center at Houston. https://med.uth.edu/harrishealth/wp-content/uploads/sites/26/2015/01/Handout_of_hemostatic_agents_for_JW_3-8-14.pdf

#### Other debriefing points

Keep the two tables with the contaminated airway model and the balloon tamponade set up in the room for the duration of the debrief. Debriefing these procedural techniques is much easier with physical demonstration. The SIM coordinator can remove both tables after the debrief session and reset them during a 5-minute transition period between groups

## SIMULATION ASSESSMENT

*Massive Upper Gastrointestinal Bleeding*

Learner: _________________________________________

***Assessment Timeline***

This timeline is to help observers assess their learners. It allows observer to make notes on when learners performed various tasks, which can help guide debriefing discussion.[Table t11-jetem-7-1-s21]

**Critical Actions:**

□ Assess airway, breathing and circulation
□ Connect the patient to the cardiopulmonary monitor and obtain large bore IV access
□ Obtain a thorough history and perform a complete physical exam
□ Assess for causes of UGIB
□ Initiate pharmacologic therapy for UGIB from suspected varices including octreotide, ceftriaxone, proton pump inhibitor, and blood
□ Order a CT head due to head trauma in a patient with alcoholic cirrhosis
□ Monitor closely for worsening bleeding by frequent clinical assessments or placing a nasogastric tube
□ Assess for airway protection in patient with worsening clinical status and establish endotracheal intubation
□ Consider at least three alternatives to blood product administration for UGIB in patient who is a Jehovah’s Witness
□ Place a gastroesophageal balloon tamponade device for massive UGIB to control the worsening bleeding
□ Consult gastroenterology and admit the patient to the intensive care unit (ICU)
□ Discuss the case with patient’s husband

**Summative and formative comments:**

**Milestones assessment:** [Table t12-jetem-7-1-s21]
