# Supplementary material for: Massive Upper Gastrointestinal Bleeding
Source: J Educ Teach Emerg Med. 2022 Jan 15;7(1):S21–50. doi: 10.21980/J8W93W (PMC10358865; doi:10.21980/J8W93W)
Supplement: Supplementary file 1 [file JETem-7-1-S21-supp1.pptx]

## Slide 1
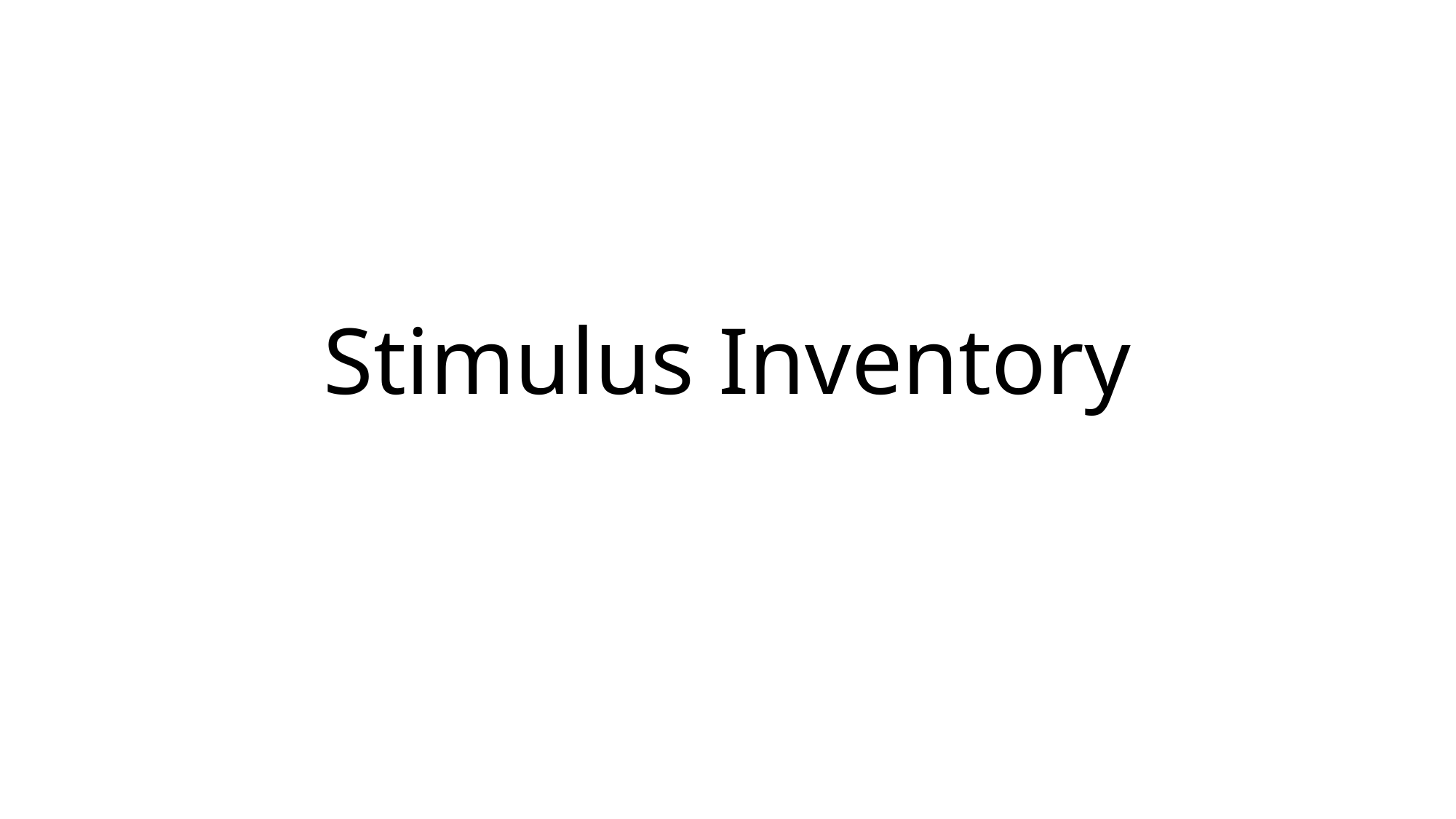

# Stimulus Inventory

## Slide 2
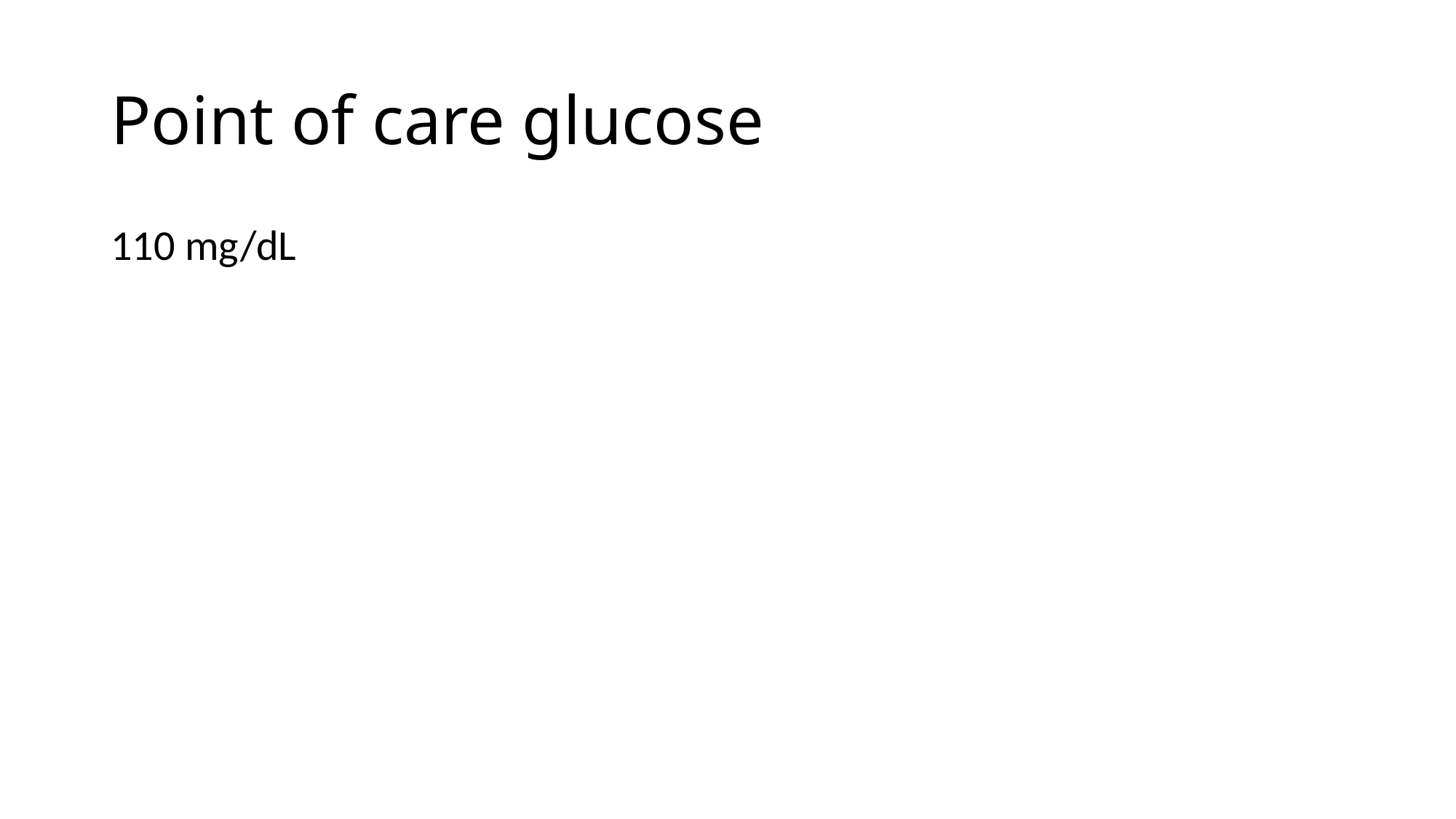

# Point of care glucose
110 mg/dL

## Slide 3
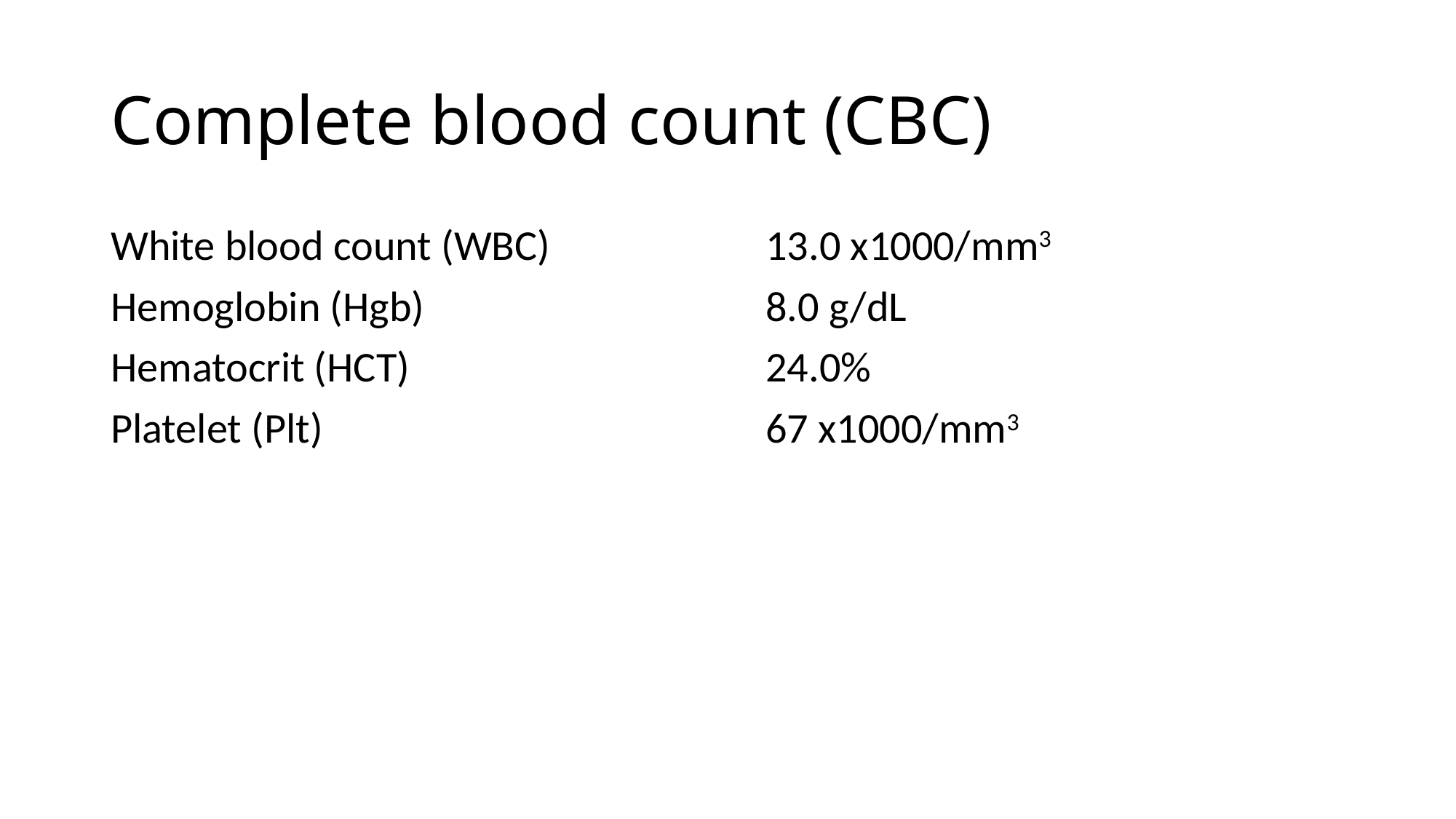

# Complete blood count (CBC)
White blood count (WBC) 		13.0 x1000/mm3
Hemoglobin (Hgb)				8.0 g/dL
Hematocrit (HCT)				24.0%
Platelet (Plt) 				67 x1000/mm3

## Slide 4
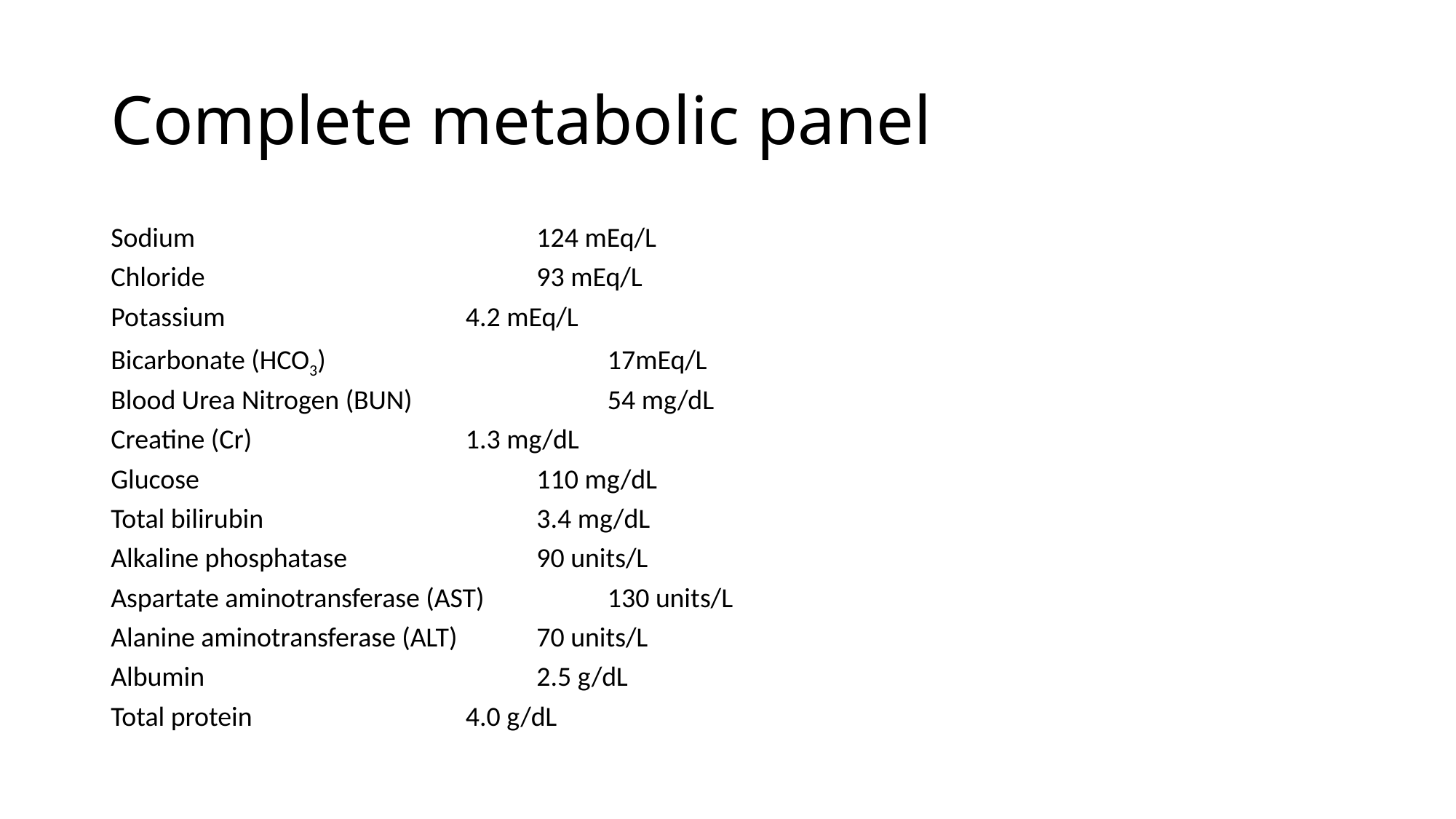

# Complete metabolic panel
Sodium 					124 mEq/L
Chloride 					93 mEq/L
Potassium				4.2 mEq/L
Bicarbonate (HCO3)				17mEq/L
Blood Urea Nitrogen (BUN)			54 mg/dL
Creatine (Cr)		 		1.3 mg/dL
Glucose 					110 mg/dL
Total bilirubin				3.4 mg/dL
Alkaline phosphatase			90 units/L
Aspartate aminotransferase (AST)		130 units/L
Alanine aminotransferase (ALT)		70 units/L
Albumin					2.5 g/dL
Total protein				4.0 g/dL

## Slide 5
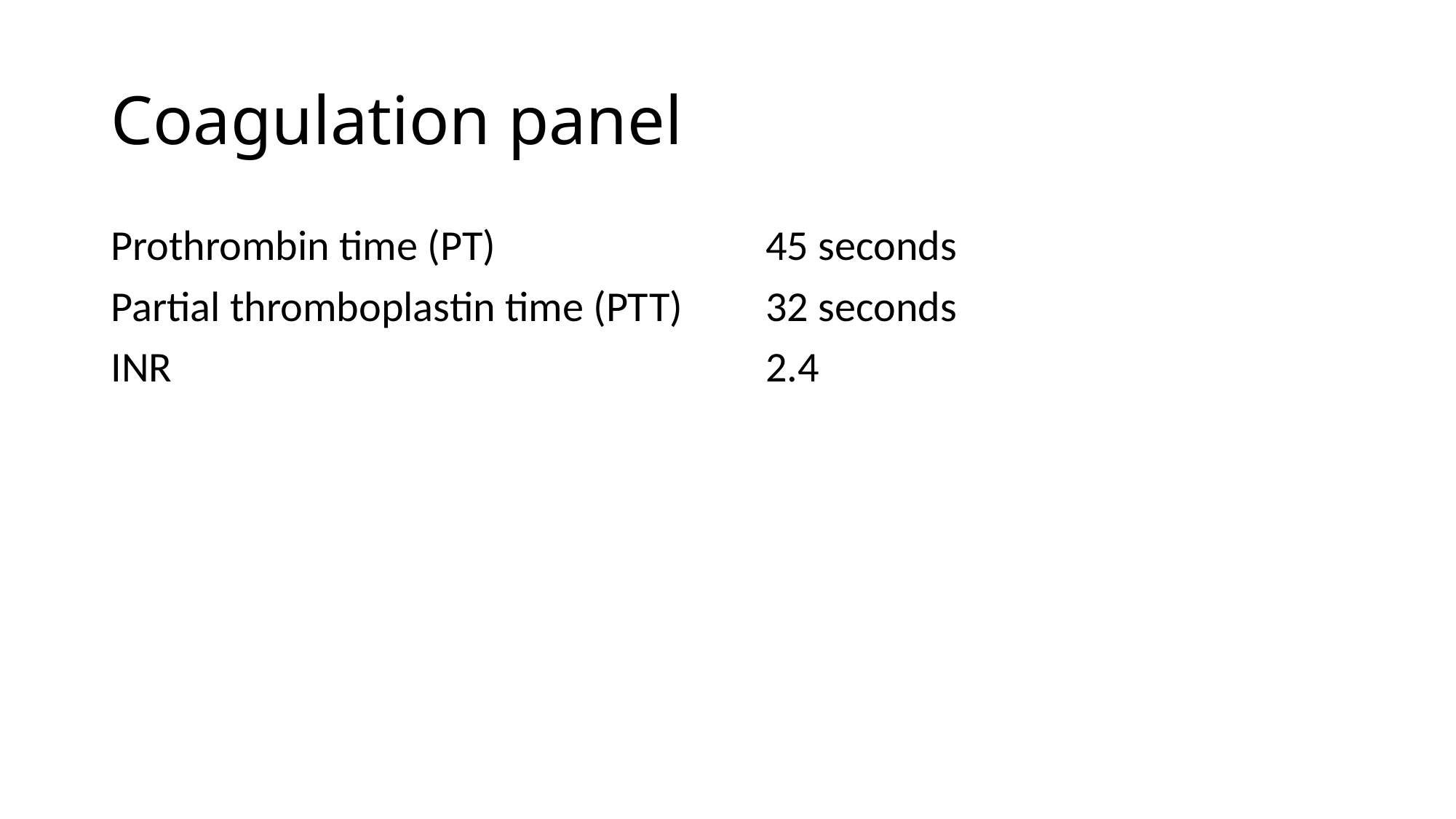

# Coagulation panel
Prothrombin time (PT) 			45 seconds
Partial thromboplastin time (PTT) 	32 seconds
INR 						2.4

## Slide 6
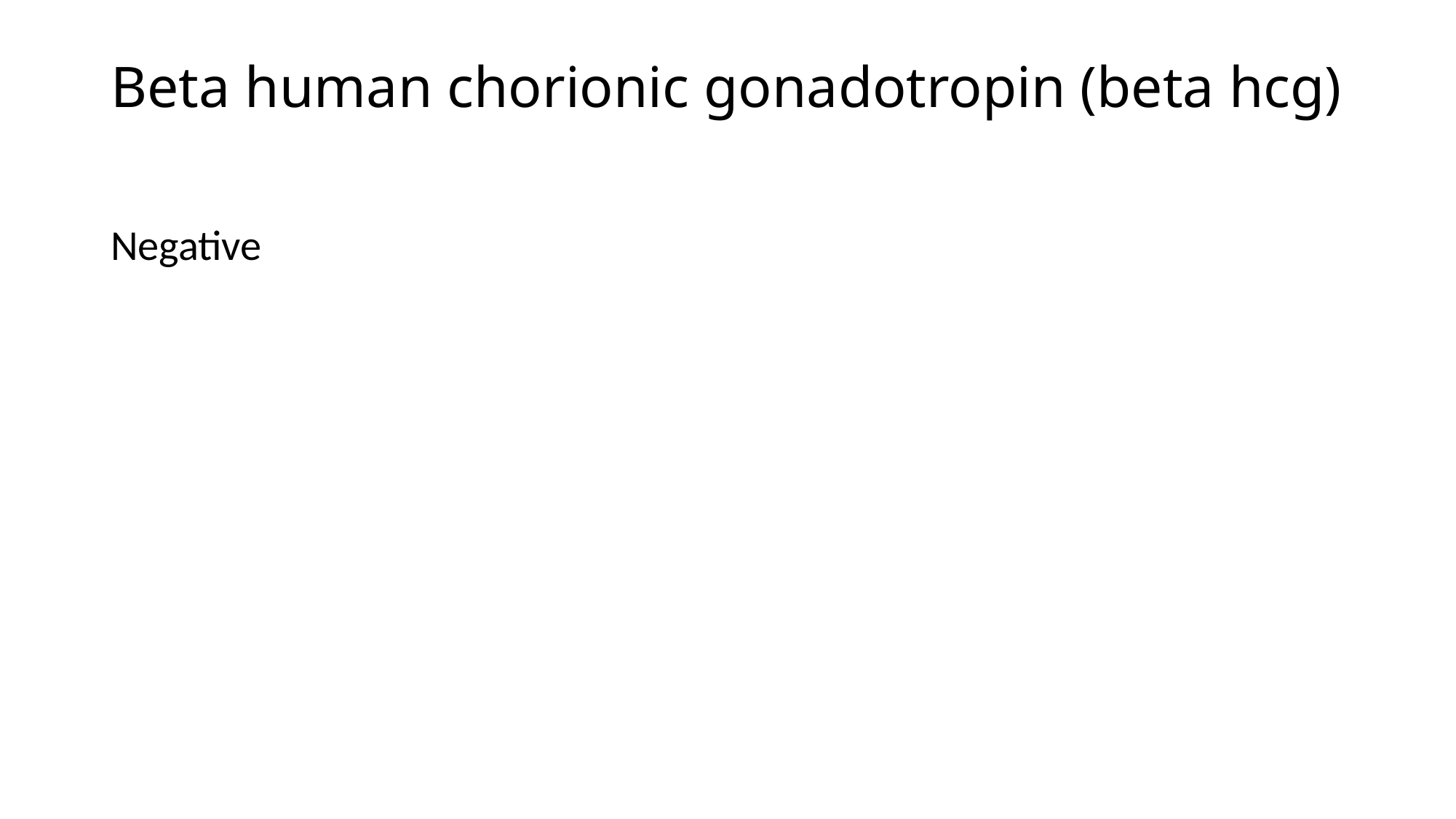

# Beta human chorionic gonadotropin (beta hcg)
Negative

## Slide 7
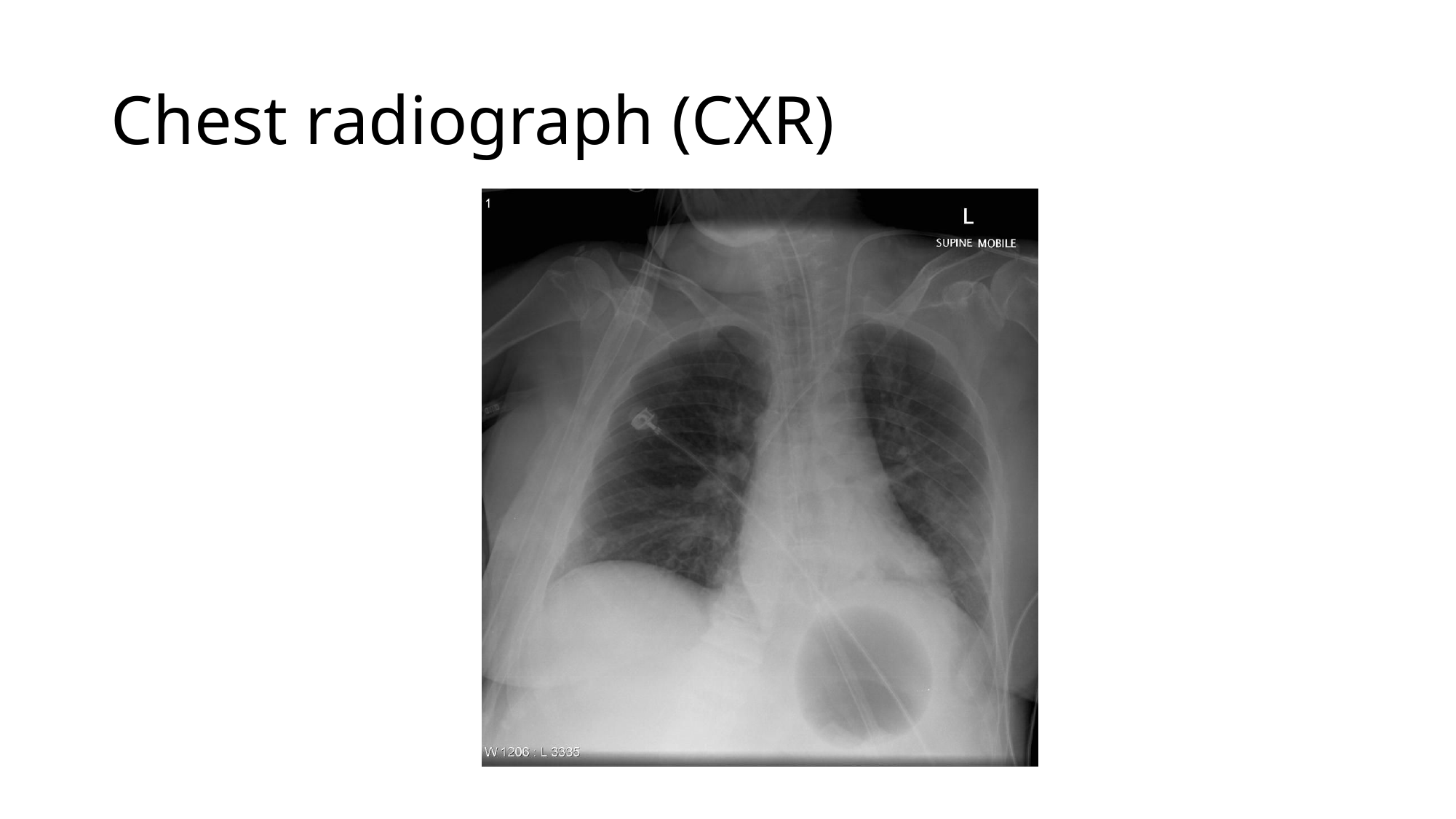

# Chest radiograph (CXR)

## Slide 8
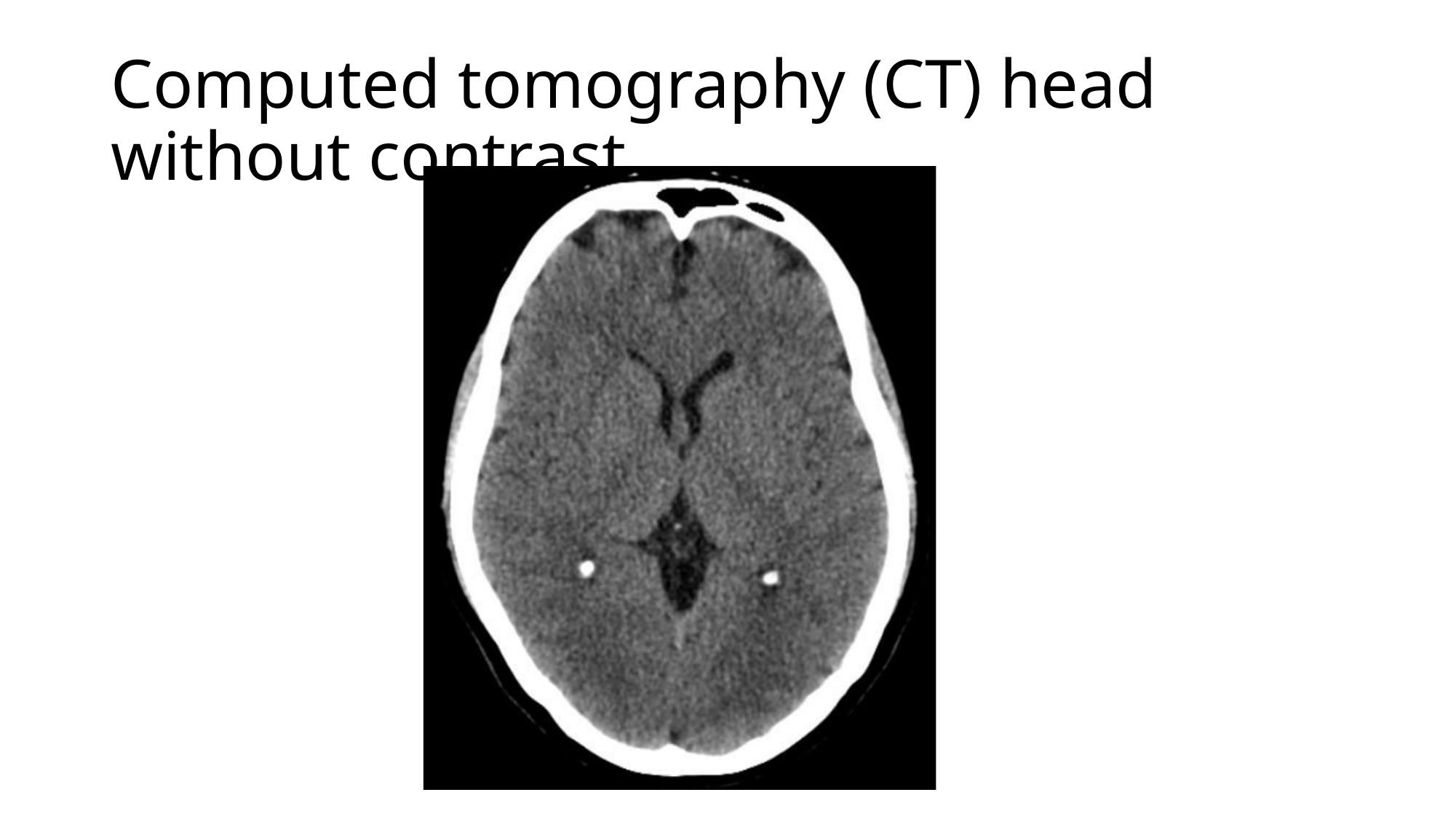

# Computed tomography (CT) head without contrast
